# The long non-coding RNA Kcnq1ot1 controls maternal *p57* expression in muscle cells by promoting H3K27me3 accumulation to an intragenic MyoD-binding region

**DOI:** 10.1186/s13072-019-0253-1

**Published:** 2019-01-16

**Authors:** Oriella Andresini, Marianna Nicoletta Rossi, Francesca Matteini, Stefano Petrai, Tiziana Santini, Rossella Maione

**Affiliations:** 1 Department of Molecular Medicine, Sapienza University of Rome, Viale Regina Elena 324, 00161 Rome, Italy; 2 Present Address: Rheumatology Unit, Bambino Gesu Children’s Hospital (IRCCS), Viale di S. Paolo 15, 00146 Rome, Italy; 3 Center for Life Nano Science@Sapienza, Istituto Italiano di Tecnologia, Viale Regina Elena 291, 00161 Rome, Italy

**Keywords:** p57^kip2^/Cdkn1c, Kcnq1ot1, Muscle differentiation, MyoD, H3K27me3, Imprinting

## Abstract

**Background:**

The cell-cycle inhibitor p57^kip2^ plays a critical role in mammalian development by coordinating cell proliferation and differentiation in many cell types. p57^kip2^ expression is finely regulated by several epigenetic mechanisms, including paternal imprinting. Kcnq1ot1, a long non-coding RNA (LncRNA), whose gene maps to the *p57*^*Kip2*^ imprinting domain, is expressed exclusively from the paternal allele and participates in the *cis*-silencing of the neighboring imprinted genes through chromatin-level regulation. In light of our previous evidence of a functional interaction between myogenic factors and imprinting control elements in the regulation of the maternal *p57*^*Kip2*^ allele during muscle differentiation, we examined the possibility that also Kcnq1ot1 could play an imprinting-independent role in the control of *p57*^*Kip2*^ expression in muscle cells.

**Results:**

We found that Kcnq1ot1 depletion by siRNA causes the upregulation of the maternal and functional *p57*^*Kip2*^ allele during differentiation, suggesting a previously undisclosed role for this LncRNA. Consistently, Chromatin Oligo-affinity Precipitation assays showed that Kcnq1ot1 physically interacts not only with the paternal imprinting control region of the locus, as already known, but also with both maternal and paternal alleles of a novel *p57*^*Kip2*^ regulatory region, located intragenically and containing two binding sites for the muscle-specific factor MyoD. Moreover, chromatin immunoprecipitation assays after Kcnq1ot1 depletion demonstrated that the LncRNA is required for the accumulation of H3K27me3, a chromatin modification catalyzed by the histone-methyl-transferase EZH2, at the maternal *p57*^*kip2*^ intragenic region. Finally, upon differentiation, the binding of MyoD to this region and its physical interaction with Kcnq1ot1, analyzed by ChIP and RNA immunoprecipitation assays, correlate with the loss of EZH2 and H3K27me3 from chromatin and with *p57*^*Kip2*^ de-repression.

**Conclusions:**

These findings highlight the existence of an imprinting-independent role of Kcnq1ot1, adding new insights into the biology of a still mysterious LncRNA. Moreover, they expand our knowledge about the molecular mechanisms underlying the tight and fine regulation of *p57*^*Kip2*^ during differentiation and, possibly, its aberrant silencing observed in several pathologic conditions.

**Electronic supplementary material:**

The online version of this article (10.1186/s13072-019-0253-1) contains supplementary material, which is available to authorized users.

## Background

The *p57*^*Kip2*^ gene (also known as *Cdkn1c* and hereafter termed *p57*) encodes a member of the Cip/Kip family of the cyclin-dependent-kinase (CDK) inhibitors. p57 is a critical regulator of cell proliferation and differentiation during mammalian development and plays unique functions respect to the other family members [1]. In addition to cell cycle, p57 also influences other cellular activities, such as cell migration, apoptosis and senescence [2, 3]. Consistent with its multiple roles in cellular and developmental processes, the abnormal function of p57 causes several types of growth-related diseases and cancer [4–7].

The regulation of *p57* expression is the object of extensive investigation. In fact, most of the human growth disorders related to p57 malfunction result from altered gene expression rather than from gene mutations. Moreover, since *p57* is an imprinted gene, its transcriptional regulation represents a paradigmatic example of epigenetic control of gene expression.

*p57* maps to the growth-related *Cdkn1c/Kcnq1* imprinted domain, which spans about 1 Mb along the distal arm of chromosome 7 in mouse and the p15.5 region of chromosome 11 in human [8]. This domain includes 10 protein-coding genes, including *p57*, which are expressed from the respective maternally derived alleles, and the non-protein-coding gene *Kcnq1ot1* (*Kcnq1* opposite transcript 1), which is transcribed antisense to the protein-coding gene *Kcnq1* into a regulatory long non-coding RNA (LncRNA) from the paternally derived allele [9]. A number of strategies based on epigenetic mechanisms cooperate to establish and maintain the silencing of the non-expressed *p57* allele. An imprinting control region, KvDMR1 (Kv-differentially methylated region 1), located about 150 kb downstream of *p57*, bears differential epigenetic marks on the two parental alleles and harbors multiple and partially overlapping regulatory elements [10–12]. Among these elements, a CTCF-dependent chromatin insulator and the promoter of *Kcnq1ot1*, which are both hypomethylated and consequently active on the paternal KvDMR1, have been clearly involved in establishing the *cis*-silencing of *p57* and of the other protein-coding genes of the imprinted domain [10, 13–15].

Consistent with the critical importance of the proper *p57* expression dosage, not only the paternal allele is ubiquitously and permanently silenced, but also the maternal allele is tightly and finely regulated during development and differentiation [16]. Our work in muscle cells revealed that KvDMR1, in addition to control the silencing of the imprinted *p57* paternal allele, is also involved in preventing the expression of the functional *p57* maternal allele until differentiation occurs [17, 18]. In particular, in undifferentiated myoblasts, KvDMR1 participates in a repressive long-range chromatin interaction with *p57* promoter, mediated by CTCF. Upon differentiation, the myogenic factor MyoD binds to specific target sequences adjacent to CTCF binding sites within KvDMR1. This interaction, which is prevented by the presence of repressive chromatin marks in myogenic cell types unable to express *p57* [19], causes the disruption of the CTCF-mediated loop and the induction of maternal *p57* expression.

Kcnq1ot1 is a macro LncRNA of about 90 kb, unspliced and exclusively localized in the nucleus [20]. Loss of paternal Kcnq1ot1, as a result of either promoter deletion [21] or premature transcript termination [15, 21], is associated with loss of imprinting. However, the molecular mechanisms by which Kcnq1ot1 contributes to gene silencing are far from being clarified, in large part due to its high complexity, resulting from the enormous size and to the consequent difficulties in every experimental approach to its study. Kcnq1ot1 has been shown to coat specific chromatin regions along the *Cdkn1c*/*Kcnq1* domain [22–24]. The pattern and the extent of Kcnq1ot1-chromatin interactions correlate with the accumulation of repressive histone modifications on the regulatory regions of imprinted genes and with their silencing [25, 26]. This is at least in part explained by the property of Kcnq1ot1 to interact with histone methyltransferases, such as G9a and the Polycomb Repressive Complex 2 (PRC2) components, recruiting them to its target genes to establish their imprinted status [23, 27]. Kcnq1ot1 also interacts with the DNA Methyl Transferase 1 (DNMT1), and this interaction is required for the accumulation of DNA methylation at the promoters of some imprinted genes and for the maintenance of their silencing [28, 29].

The molecular mechanisms by which Kcnq1ot1 associates with non-overlapping target genes, spread over almost 1 Mb, are not clear. It is believed that LncRNAs can localize to specific DNA regions through affinity interactions with local factors, such as chromatin proteins and transcription factors and/or through the establishment of physical contacts between distant genomic regions resulting from chromatin looping [30, 31]. Regarding Kcnq1ot1, it has been shown that the LncRNA itself participates in the establishment of higher order intra-chromosomal interactions [32]. Moreover, it is becoming increasingly apparent that LncRNAs, including Kcnq1ot1, can target specific DNA sequences by forming RNA–DNA triplexes [33].

Another unresolved question concerns the molecular mechanisms by which Kcnq1ot1, as well as other LncRNAs, associates with and organize the regulatory complexes recruited on its targets. It has been reported that a conserved 890 bp sequence, mapping at the 5’ of Kcnq1ot1 and termed silencing domain, is required for the interaction of the LncRNA with DNMT1 and for its association with chromatin [28]. However, it is still unclear if this sequence mediates a direct or an indirect interaction of Kcnq1ot1 with chromatin and/or with DNMT1 or if it is involved in the formation of a crucial structure of the LncRNA.

Even less is known about the regulation of Kcnq1ot1 activity. *Kcnq1ot1* is expressed at relatively high levels in all tissues analyzed [9], but its pattern of chromatin binding and its ability to interact with histone modifying complexes are different between tissues [23]. This suggests that the target specificity of Kcnq1ot1-mediated silencing can be modulated by additional, and likely complex, regulatory interactions.

The little information available to date on the function of Kcnq1ot1 in *p57* regulation concerns, almost exclusively, the imprinting control. In light of our previous evidence that some of the factors involved in the regulation of the paternal allele during imprinting, such as CTCF and KvDMR1, can be also involved in the regulation of the maternal allele during differentiation, we wanted to examine the possibility that Kcnq1ot1 could play an imprinting-independent role in the MyoD-dependent regulation of *p57* during differentiation.

In the present work, we show that an additional level of transcriptional regulation of maternal *p57* in muscle cells involves the functional interaction of Kcnq1ot1 with a novel and intragenic regulatory region of the *p57* gene. This level of regulation implies the Kcnq1ot1-dependent accumulation of the repressive histone modification H3K27me3 in undifferentiated cells and its loss upon differentiation, correlated with the interaction of MyoD with Kcnq1ot1 at the same region.

## Results

### Kcnq1ot1 depletion causes the upregulation of the *p57* maternal allele

**Fig. 1:**
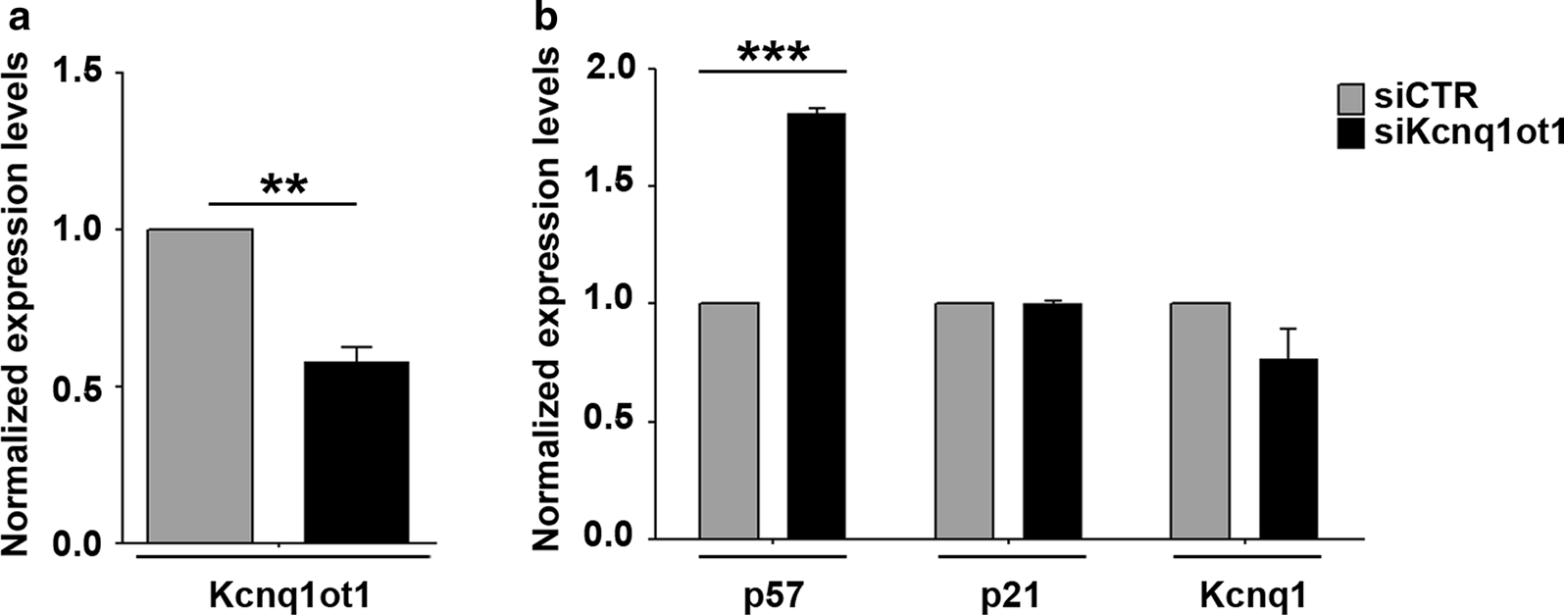
Kcnq1ot1 knockdown boosts *p57* induction in differentiating myoblasts. **a** Proliferating C2.7 myoblasts were transfected with Kcnq1ot1 siRNAs. After 24 h cells were collected to analyze Kcnq1ot1 levels by RT-qPCR in samples depleted for Kcnq1ot1 transcript (siKcnq1ot1) compared to the control (siCTR). Values, relative to those of Tbp RNA, are the mean ± SEM of three independent experiments. Statistical significance: *p* value < 0.01 (**); **b** RT-qPCR analysis of *p57*, *p21* and *Kcnq1* expression in siCTR and siKcnq1ot1-transfected cells 24 h after the shift to differentiation medium. Values, relative to those of Tbp RNA, are the mean ± SEM of three independent experiments. Statistical significance: *p* value < 0.001 (***)

We previously reported that repressive epigenetic changes at *p57* promoter and KvDMR1 are involved in the transcriptional control of the gene during muscle differentiation [18, 19, 34]. Since Kcnq1ot1, by recruiting chromatin modifiers, is capable to establish epigenetic marks at least on the paternal *p57* allele [23], we asked whether the LncRNA could also mediate the epigenetic regulation of the maternal *p57* allele in muscle cells. In light of the demonstrated ability of siRNAs and shRNAs to efficiently deplete nuclear transcripts [35], including Kcnq1ot1 [36–38], we performed a knockdown assay to assess the effects of Kcnq1ot1 depletion on *p57* expression. Proliferating myoblasts were transfected with a Kcnq1ot1-targeting pool of small-interfering RNAs and a non-targeting pool as a control. Kcnq1ot1 depletion was verified through RT-qPCR assays. As shown in Fig. 1a, Kcnq1ot1 transcript levels were efficiently reduced respect to the control. The expression levels of *p57* were analyzed by collecting samples 24 h after the shift to differentiation medium, a condition that allows MyoD activation and *p57* induction. Interestingly, as shown in Fig. 1b, Kcnq1ot1 depletion correlates with a significant increase of *p57* expression. In contrast, the expression of *p21*, a related CDK inhibitor and a MyoD target as well [39], was not changed at all. Unexpectedly, even the expression of *Kcnq1*, which is overlapped in antisense orientation by *Kcnq1ot1* [9, 40], was not increased by the depletion of the LncRNA, suggesting that the observed effect on gene expression is specific for *p57*.

**Fig. 2:**
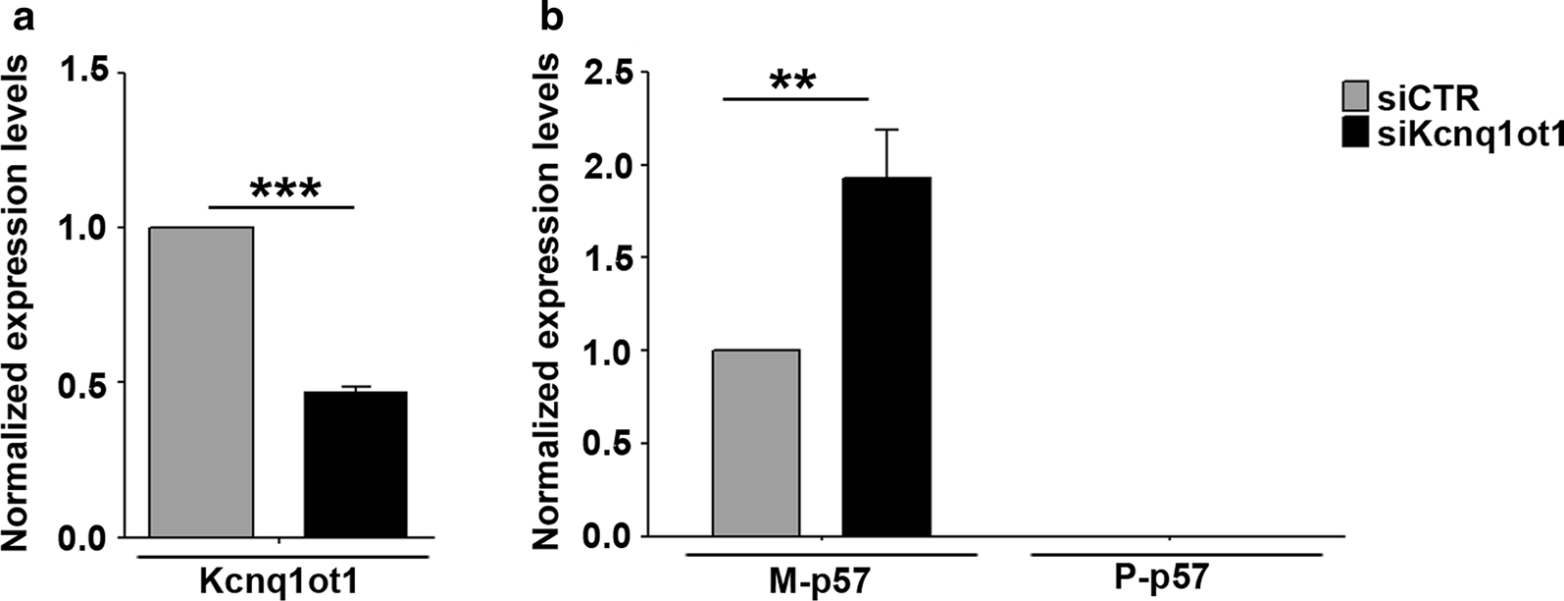
Kcnq1ot1 knockdown affects maternal but not paternal *p57* expression. **a** Polymorphic fibroblasts (C57B/6 female × SD7 male) infected with the MyoD retroviral vector were transfected with Kcnq1ot1 or control siRNAs. After 24 h, cells were collected to analyze Kcnq1ot1 levels by RT-qPCR in samples depleted for Kcnq1ot1 transcript (siKcnq1ot1) compared to the control (siCTR). Values, relative to those of Tbp RNA, are the mean ± SEM of three independent experiments. Statistical significance: *p* value < 0.001 (***); **b** Allele-specific RT-qPCR analysis of *p57* (M-*p57*: Maternal *p57*; P-*p57*: Paternal *p57*) expression was performed in siCTR and siKcnq1ot1 samples using allele-specific primers. Values, relative to those of Tbp RNA, are the mean ±SEM of three independent experiments. Statistical significance: *p* value < 0.01 (**)

Since Kcnq1ot1 participates in the silencing of the imprinted *p57* paternal allele, we asked whether the increased *p57* expression, observed after Kcnq1ot1 depletion, was due to loss of imprinting or rather to the upregulation of the active, maternal *p57* allele. To investigate this issue, we took advantage of a muscle differentiation system based on the myogenic conversion of non-muscle cells by exogenous MyoD expression. This system gave us the opportunity to exploit mouse fibroblasts carrying single nucleotide polymorphisms in the *p57* locus in order to examine allele-specific expression during myogenesis [17–19]. Cells were infected with a MyoD retroviral vector and cultured in proliferation medium so as to prevent differentiation up to the time of Kcnq1ot1 knockdown. After 48 h cells were transfected with Kcnq1ot1 or control siRNA pools. 24 h later cells were either collected, to confirm Kcnq1ot1 downregulation (Fig. 2a) or shifted to differentiation medium, to allow the induction of MyoD targets. As reported in Additional file 1, Kcnq1ot1 downregulation correlates with *p57*, but not *p21* upregulation also in MyoD-converted fibroblasts, just like in myoblasts. The contribution of the maternal and/or paternal alleles to the increase of *p57* expression upon Kcnq1ot1 depletion was investigated by allele-specific RT-qPCR, using primers allowing us to measure specifically, as well as quantitatively, maternal and paternal p57 mRNA levels. As shown in Fig. 2b, the increased levels of *p57* after Kcnq1ot1 depletion are exclusively accounted for by the increased expression of maternal *p57*, and not to the re-expression of the imprinted paternal allele. Allele-specific analysis was also performed by restriction fragment length polymorphism (RFLP) assays. The results reported in Additional file 2 confirmed that the increased expression of *p57* caused by Kcnq1ot1 depletion does not involve loss of imprinting. These results indicate that the presence of Kcnq1ot1 constrains the expression of maternal *p57* during muscle differentiation.

### Kcnq1ot1 interacts with a *p57* intragenic region at both maternal and paternal alleles

The expression of *Kcnq1ot1*, unlike that of *p57* and of the co-imprinted gene *Kcnq1*, does not decrease but, rather, slightly increases in differentiating C2.7 muscle cells [18] (Additional file 3). This finding rules out a simple model where the upregulation of *p57* would be linked with a differentiation-dependent decline of *Kcnq1ot1* expression. Considering that the activities of LncRNAs are not necessarily regulated by their expression levels, but often depend on their differential interaction with chromatin or with protein complexes, we first explored whether Kcnq1ot1 was able to interact with the chromatin at the *p57* locus.

**Fig. 3:**
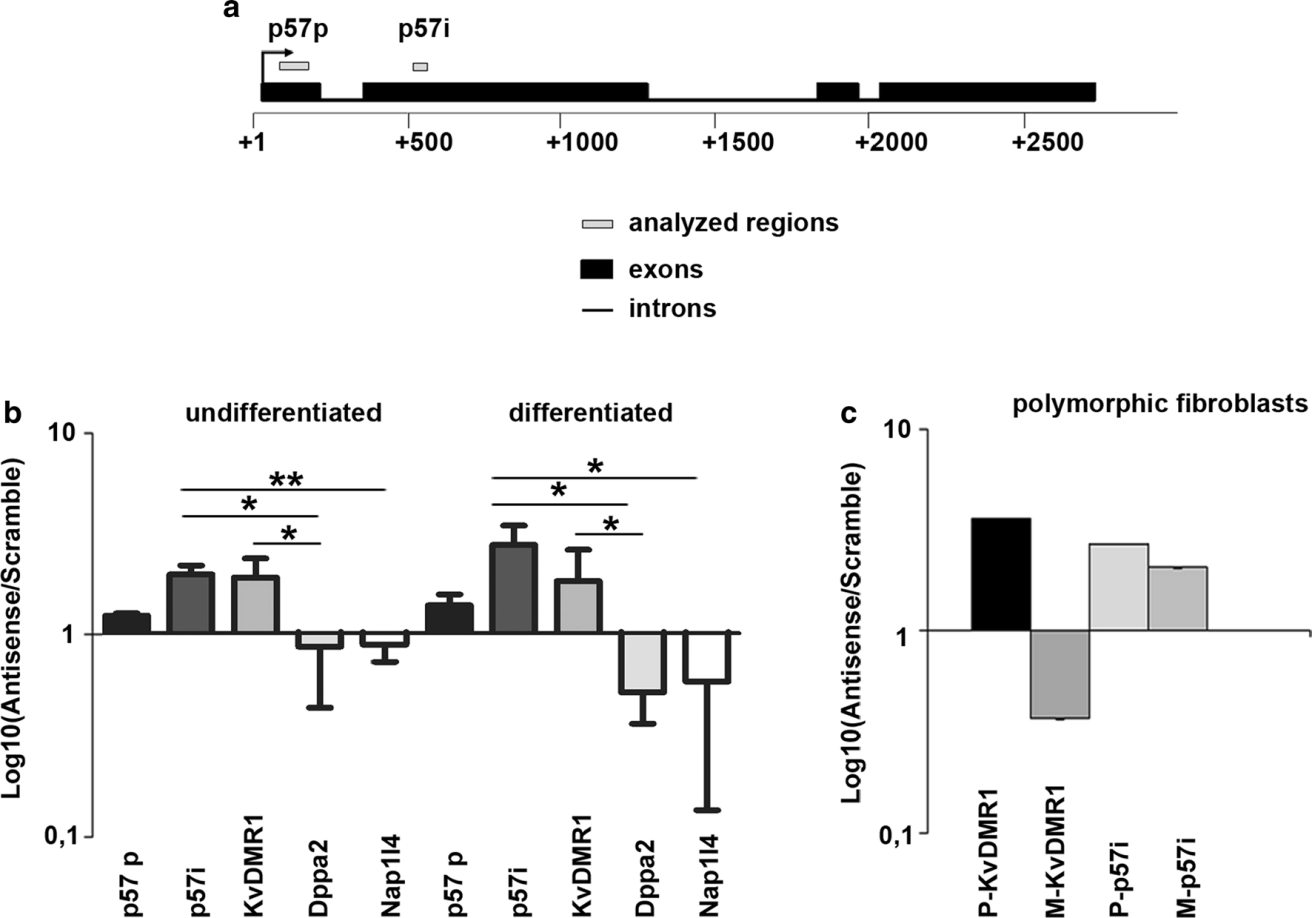
Kcnq1ot1 constitutively interacts with an intragenic region of the maternal *p57* allele. **a** Schematic representation of the *p57* gene; black blocks represent exons, black lines introns and gray boxes the regions analyzed for Kcnq1ot1 binding; *p57* p indicates the *p57* promoter and *p57i* the *p57* intragenic region. **b** ChOP assays performed in undifferentiated and differentiated C2.7 muscle cells; qPCR analysis of purified DNA performed using primers specific for *p57* p, *p57*i and KvDMR1. *Dppa2* and Nap1l4 were used as negative controls. The enrichment with antisense oligonucleotides over scrambled oligonucleotides was plotted in logarithmic scale. Data represent the mean ± SEM of three independent experiments. Statistical significance: *p* < 0.05 (*). **c** ChOP assays performed in polymorphic fibroblasts (C57B/6 female × SD7 male); qPCR analysis of purified DNA was performed using allele-specific primers for KvDMR1 (M-KvDMR1: Maternal KvDMR1; P-KvDMR1: Paternal KvDMR1) and *p57*i (M-*p57i*: Maternal *p57i*; P-*p57i*: Paternal *p57i*). Values represent one of two independent experiments, and error bars represent the mean ± SEM of each sample analyzed in triplicate

For this purpose, we performed Chromatin Oligo-affinity Precipitation (ChOP) assays [23] in C2.7 muscle cells. We focused our attention not only on *p57* promoter, but also on an intragenic region located in the second exon of the gene (*p57i*) that, by inspecting the ChIP-seq data for muscle cells from ENCODE/Caltech, showed increased levels of histone H3 acetylation upon differentiation, suggesting its potential regulatory role. Kcnq1ot1 RNA-associated-chromatin was affinity-purified using either a biotin-labeled antisense oligonucleotide against the Kcnq1ot1 transcript or a biotin-labeled scrambled oligonucleotide as a control. qPCR analysis was performed on purified DNA samples using primers corresponding to the regions of interest as outlined in Fig. 3a. As expected, and as shown in Fig. 3b, we observed that Kcnq1ot1 interacts with the KvDMR1 region from which it is transcribed. Remarkably, as reported in the same figure, we also found a significant interaction of Kcnq1ot1 with the *p57i*. Some enrichment, although not statistically significant, was also detectable for *p57* promoter. In contrast, no enrichment was observed for the promoter of *Nap1l4* (*Nucleosome Assembly Protein 1 Like 4*), which maps nearby the imprinting domain, about 90 kb upstream of p57, nor for the promoter of *Dppa2* (*Developmental pluripotency associated 2*), which is located on chromosome 16. To discriminate the allele-specific interactions of Kcnq1ot1 with KvDMR1 and with the *p57i*, we performed ChOP assays in polymorphic fibroblasts expressing MyoD, using allele-specific primers for qPCR analysis. As shown in Fig. 3c, the Kcnq1ot1 interaction with KvDMR1 occurs only at the paternal allele, as previously reported [32]. Remarkably, unlike for KvDMR1, the interaction of Kcnq1ot1 with the *p57i* region occurs not only at the paternal allele, as it would have been expected, but also at the maternal counterpart.

**Fig. 4:**
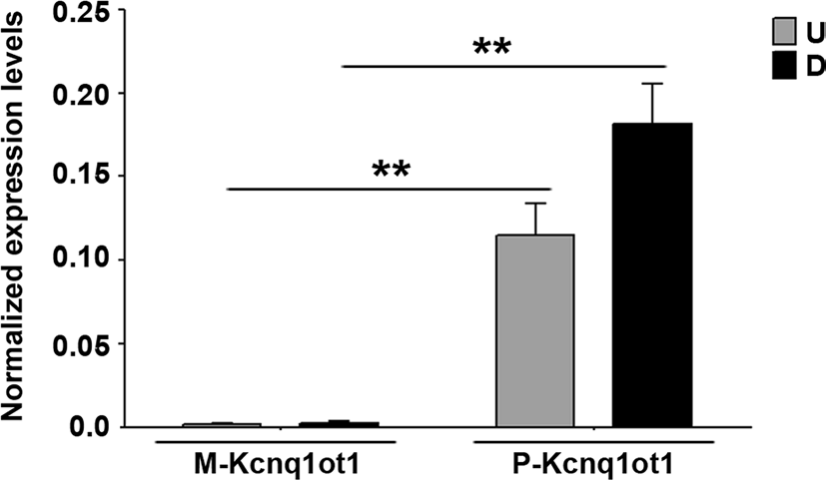
Kcnq1ot1 expression is restricted to the paternal allele and does not decrease during differentiation. Allele-specific RT-qPCR analysis of maternal and paternal Kcnq1ot1 (M-Kcnq1ot1 and P-Kcnq1ot1, respectively) performed in polymorphic fibroblasts (C57B/6 female × SD7 male) infected with the MyoD retroviral vector and analyzed in undifferentiated (*U*) and differentiated (*D*) cells. Values, normalized to Tbp expression, are the mean ± SEM of four independent experiments. Statistical significance: *p* value < 0.01 (**)

As mentioned above, *Kcnq1ot1* is thought to be expressed from the paternal allele and to act *in cis* on the same chromosome from which it is transcribed, in order to silence imprinted genes. Therefore, it was surprising to observe that Kcnq1ot1 physically interacts with the maternal *p57i* and that its knockdown affects the expression of maternal *p57*. We asked whether these findings indicated a *trans*-effect of paternally expressed Kcnq1ot1 on maternal *p57* regulatory region, or reflected the occurrence of maternal *Kcnq1ot1* expression, due to relaxation of imprinting in muscle cells. To address this issue, polymorphic fibroblasts expressing exogenous MyoD were analyzed for the relative amounts of maternal and paternal Kcnq1ot1 transcripts, by allele-specific RT-qPCR. As reported in Fig. 4, *Kcnq1ot1* expression is restricted exclusively to the paternal allele both in undifferentiated and in differentiated cells.

These and the above results, taken together, suggested that the Kcnq1ot1-associated intragenic region is a novel regulatory element affecting the functional *p57* allele in muscle cells through a mechanism involving a trans-effect of Kcnq1ot1 (see Discussion).

### Kcnq1ot1 mediates the accumulation of EZH2 and H3K27me3 at the *p57* intragenic region in undifferentiated cells

Since the interaction of Kcnq1ot1 with the *p57i* occurs both in undifferentiated and differentiated cells (Fig. 3b), we supposed that the LncRNA could function as a molecular scaffold modulating the targeting of repressive histone modifications to the maternal allele during differentiation. As mentioned above, Kcnq1ot1 interacts with several histone and DNA methyltransferases to establish the silencing of the *p57* domain during imprinting. In light of several studies showing that *p57* is a target of EZH2, the PRC2 catalytic subunit, in different cancer cell types [41–43] and in differentiating Schwann cells [44], we focused our attention on the tri-methylation of lysine 27 in histone H3 (H3K27me3), the modification catalyzed by this complex.

**Fig. 5:**
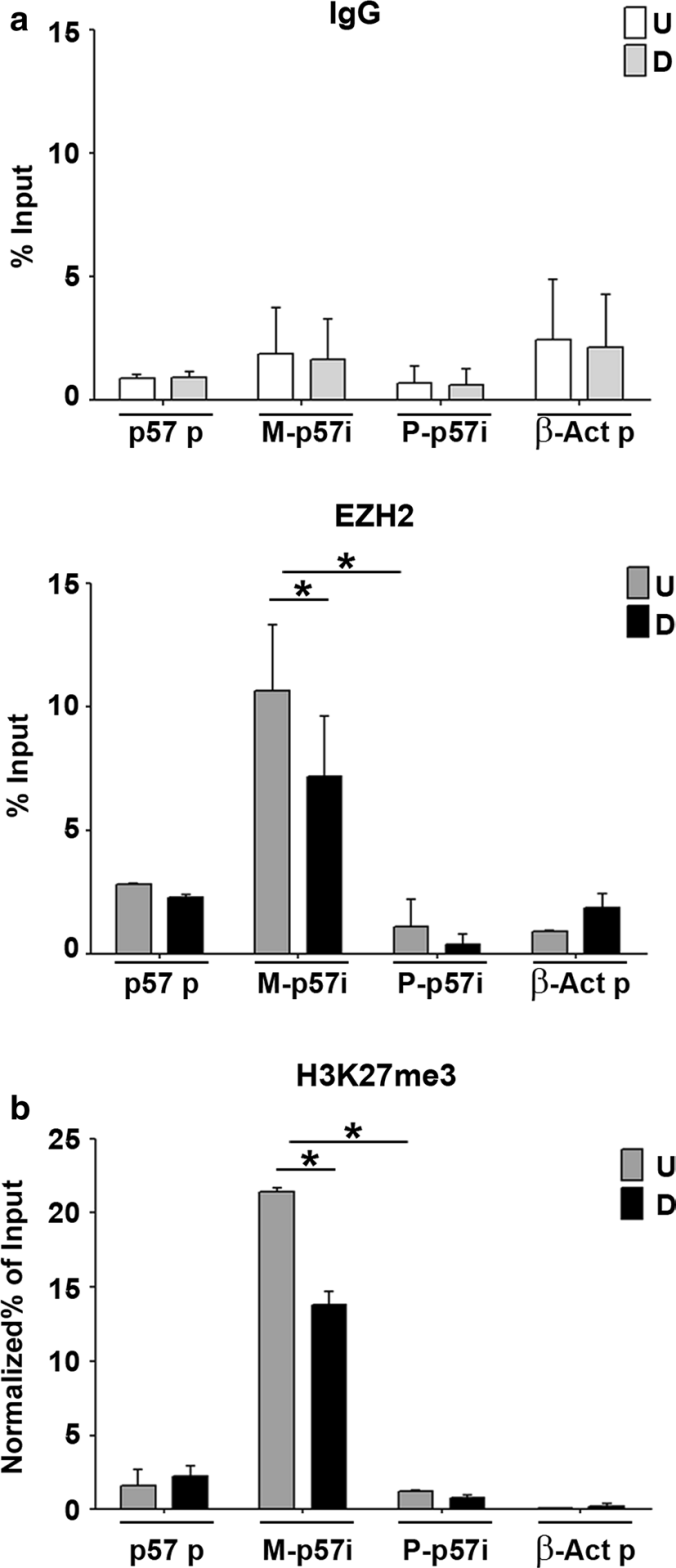
EZH2 and H3K27me3 association to the maternal *p57* intragenic region decreases during differentiation. ChIP-qPCR analysis for IgG and EZH2 (**a**) and H3K27me3 (**b**) during differentiation (*U* undifferentiated and *D* differentiated) of polymorphic fibroblasts (C57B/6 female × SD7 male) expressing exogenous MyoD. qPCR analysis was performed using primers specific for *p57* promoter (*p57* p), for maternal and paternal *p57* intragenic regions (M-*p57i* and P-*p57*i, respectively), and for β-Actin promoter (β-Act p) used as a negative control. Values are the mean of two independent experiments and expressed as percentages of Input. Values for H3K27me3 association were also normalized to those of the invariant internal control, the *Amylase* promoter. Error bars indicate the SEM. *p* value < 0.05 (*)

To explore the possible role of PRC2 activity in the Kcnq1ot1-dependent regulation of maternal *p57*, we first performed ChIP assays for EZH2 and for the H3K27me3 mark, indicative of EZH2 activity, using polymorphic fibroblasts. qPCR analysis was performed with primers specific for *p57* promoter and for the maternal and paternal *p57* intragenic regions. Interestingly, as shown in Fig. 5, EZH2, as well as H3K27me3, are associated not only with *p57* promoter, as previously reported [25, 26, 43, 44] but also, and even more significantly, with *p57i*. Remarkably, the accumulation of EZH2 and H3K27me3 on this region concerns almost exclusively the maternal allele. The presence of lower levels of H3K27me3 at the paternal *p57* allele, which is silenced, compared to the maternal *p57* allele, which is active, at first sight appears as an inconsistency. However, several lines of evidence indicate that it is DNA methylation that plays the main role in the maintenance of *p57* imprinting [45–47]. Accordingly, we found that the paternal *p57i* is significantly hypermethylated with respect to the maternal counterpart (Additional file 4). Moreover, the maternal intragenic region shows the simultaneous presence of H3K27me3 (Fig. 5) and histone H3 lysine 4 tri-methylation (H3K4me3) (Additional file 4), a chromatin profile frequently associated with intragenic CpG islands of inducible genes [48].

**Fig. 6:**
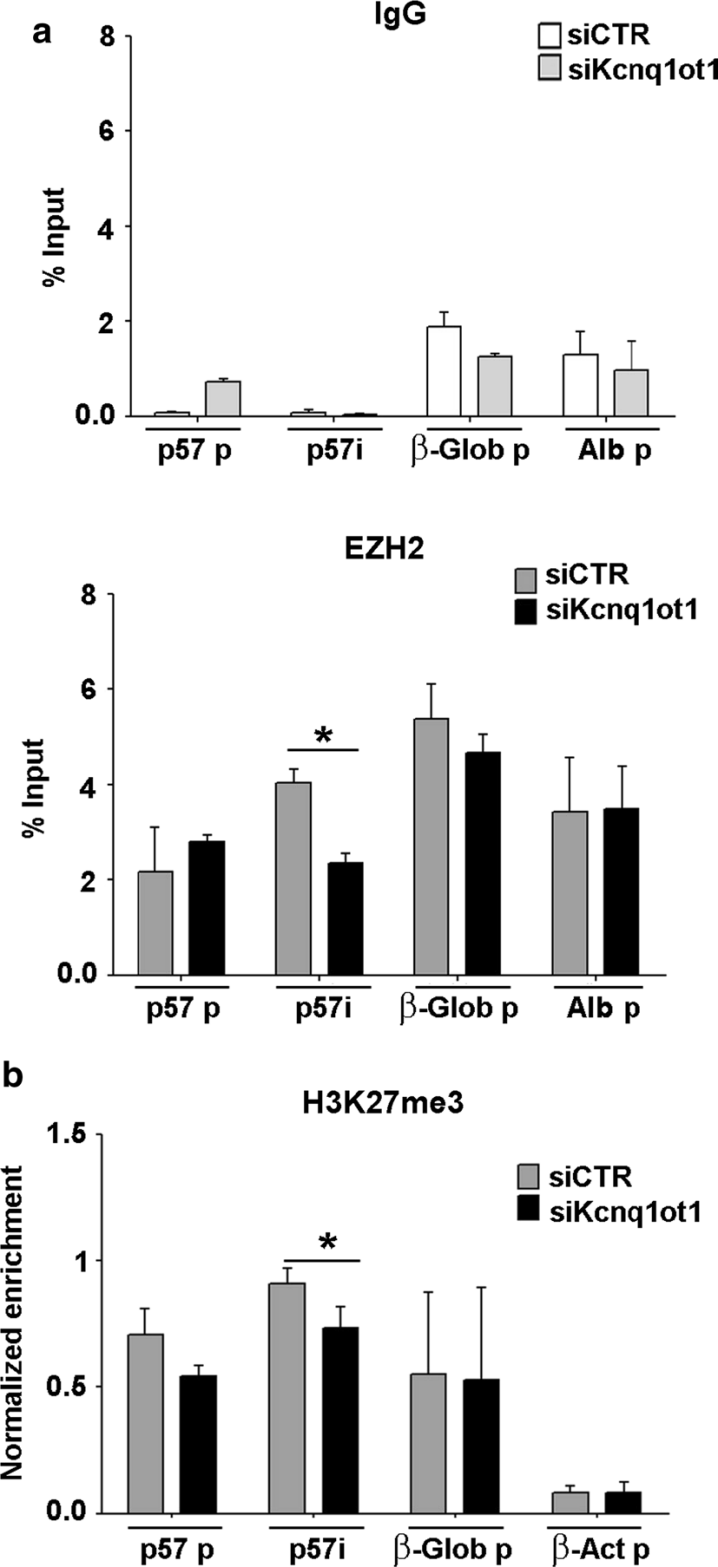
EZH2 and H3K27me3 association to the *p57* intragenic region decreases after Kcnq1ot1 depletion. C2.7 myoblasts were transfected with Kcnq1ot1 or control siRNAs as in Fig. 1a and analyzed by RT-qPCR (Additional file 5) or ChIP-qPCR. **a** ChIP-qPCR analysis for control IgG (upper panel) and for EZH2 binding (lower panel) to the *p57* promoter (*p57* p) and to the *p57* intragenic region (*p57i*). *β*-*Globin* promoter (*β*-*Glob* p) and *Albumin* promoter (*Alb* p) were used as invariant controls, **b** ChIP-qPCR analysis of H3K27me3 association to the *p57* promoter (*p57* p), *p57* intragenic region (*p57i*), *β*-*Globin* promoter (*β*-*Glob* p) used as an invariant control and β-Actin promoter (β-Act p) as a negative control. Values obtained are expressed as percentages of Input chromatin and normalized to those of *Albumin* promoter, used as an additional invariant control. Results are the mean ± SEM of three independent experiments. Statistical significance *p* value < 0.05 (*)

To prove our starting hypothesis that the EZH2-mediated association of H3K27me3 with the *p57i* requires the presence of Kcnq1ot1 as a scaffold and/or guide, we performed ChIP assays in C2.7 muscle cells after depletion of the Kcnq1ot1 transcript. Proliferating myoblasts were transfected with Kcnq1ot1 small-interfering RNAs as described above and the depletion of the LncRNA was verified through RT-qPCR analysis as reported in Additional file 5. As shown in Fig. 6a, the reduction of Kcnq1ot1 levels correlates with a significant reduction of EZH2 association with the *p57i*, but not with *β*-*globin*, *albumin* nor *p57* promoters. A parallel decrease, modest but statistically significant, was observed for H3K27me3 levels at *p57i*. A slight reduction of the histone modification was also detectable at *p57* promoter but, due to a large variability between experiments, did not result statistically significant. A similar loss of H3K27me3 accumulation at the *p57i* was observed after Knq1ot1 depletion in polymorphic fibroblasts (Additional file 6). These findings support the conclusion that the Kcnq1ot1-dependent recruitment of EZH2 promotes the repressive histone modification at the *p57i*.

Of note, the association of both EZH2 and H3K27me3 with the maternal intragenic region but not with *p57* promoter, decreases during differentiation (Fig. 5), further reinforcing the idea that it is the *p57i*, more than *p57* promoter, that plays a role in the H3K27me3-mediated regulation of *p57* during muscle differentiation. The same pattern, characterized by the association of H3K27me3 with the *p57i* and its decline in parallel with the induction of *p57*, occurs in differentiating C2.7 cells (Additional file 7).

These results, taken together, suggest that Kcnq1ot1 participates in the repression of maternal *p57* expression in undifferentiated muscle cells by influencing H3K27me3 accumulation on the *p57i* and that the induction of *p57* during differentiation correlates with the loss of this mark at this newly identified regulatory region.

### MyoD binds to the maternal *p57* intragenic region and interacts with Kcnq1ot1 in differentiated cells

**Fig. 7:**
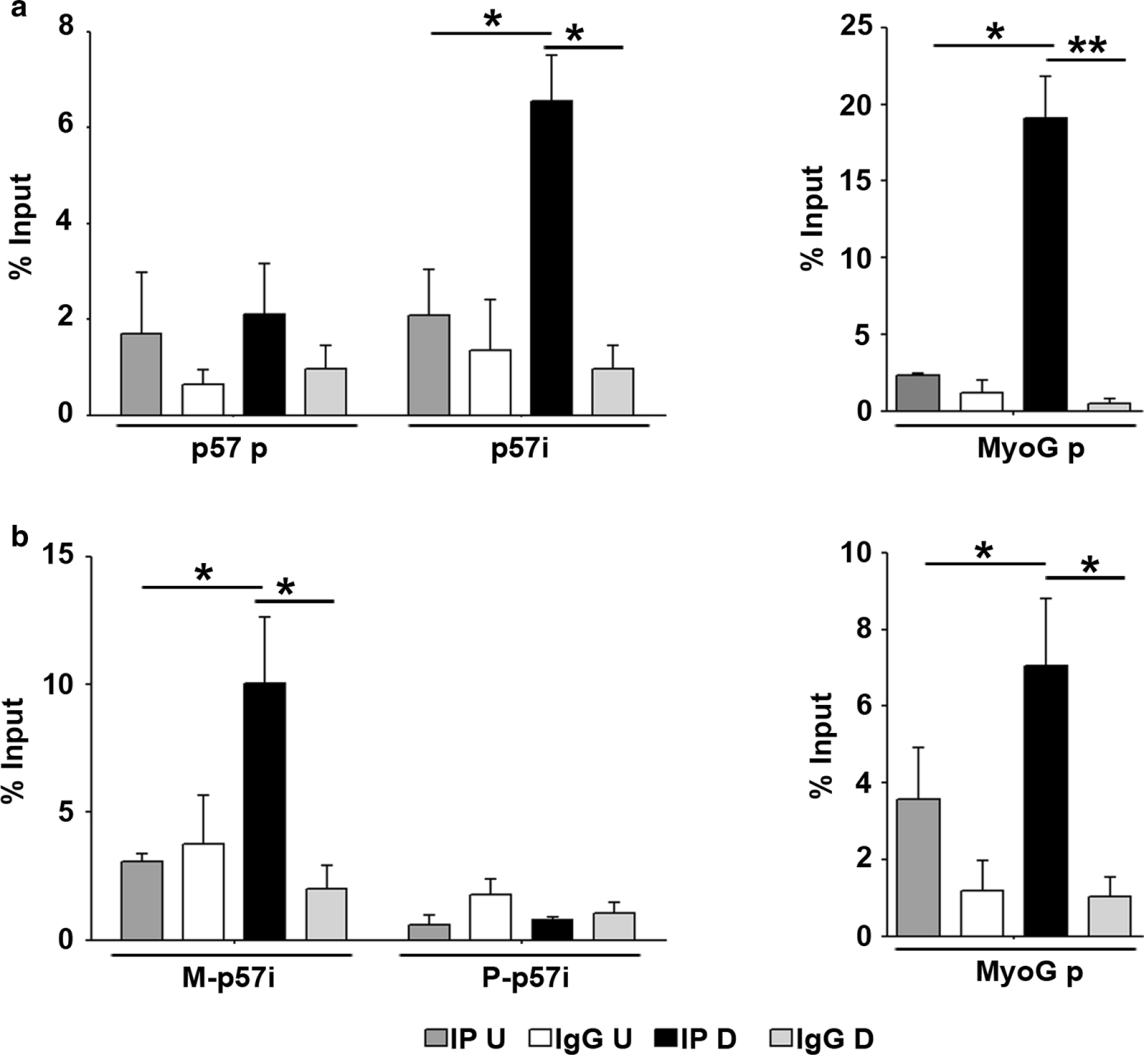
MyoD binds to the maternal *p57* intragenic region upon differentiation. **a** ChIP-qPCR analysis of MyoD binding in undifferentiated (*U*) and differentiated (*D*) C2.7 muscle cells using primers for *p57* promoter (*p57* p) and *p57* intragenic region (*p57i*) (left) and for *Myogenin* promoter (*MyoG* p) (right) used as a positive control for MyoD binding during differentiation. **b** Allele-specific ChIP-qPCR of MyoD binding in polymorphic fibroblasts (C57B/6 female × SD7 male) expressing exogenous MyoD in undifferentiated (*U*) and differentiated (*D*) cells, using primers specific for *p57* promoter (*p57* p), for maternal and paternal *p57* intragenic regions (M-*p57i* and P-*p57i*, respectively) and for *Myogenin* promoter (*MyoG* p). Values were expressed as percentages of Input and error bars indicate SEM of three independent experiments performed, excepted for the part B-left of the figure in which values are the mean ± SEM of four independent experiments. Statistical significance: *p* value < 0.05 (*) and < 0.01 (**)

It has been previously reported that EZH2 represses muscle differentiation at least in part by counteracting MyoD binding, via H3K27me3 accumulation, to muscle-specific gene promoters [49]. Using MatInspector software to scan putative MyoD-binding sites within the *p57* gene, we detected the presence of two adjacent canonical E-box sites, just in the intragenic region that we have found to be bound by Kcnq1ot1 and by EZH2. To assess the interaction of MyoD with chromatin at this region, we performed ChIP assays in C2.7 muscle cells during differentiation. The results were analyzed by qPCR using primers surrounding the putative MyoD-binding sites at the *p57i* or targeted to *p57* promoter, where we had previously demonstrated that MyoD does not bind [18]. As shown in Fig. 7a, MyoD interacts with the *p57i* and not, as expected, with *p57* promoter. Moreover, although MyoD is also expressed in undifferentiated cells [50–52], this interaction takes place only after differentiation, just like we observed for the promoter of *Myogenin*, a muscle-specific MyoD target [53]. In order to determine the allele-specificity of MyoD binding, we performed ChIP assays in polymorphic fibroblasts expressing the myogenic factor and analyzed the MyoD-immunoprecipitated chromatin through allele-specific qPCR. Remarkably, as shown in Fig. 7b, MyoD binding occurs only to the maternal allele of the *p57i* and, also in this case, only in differentiated cells. The recruitment of MyoD to the maternal allele upon differentiation reflects exactly the loss of EZH2 and H3K27me3 from the same region. On the other hand, the inability of MyoD to bind to the paternal *p57i*, where H3K27me3 levels are constitutively low, is likely a consequence of the allele-specific DNA hypermethylation of the region (Additional file 4) which renders the E-boxes permanently inaccessible to transcription factor binding.

**Fig. 8:**
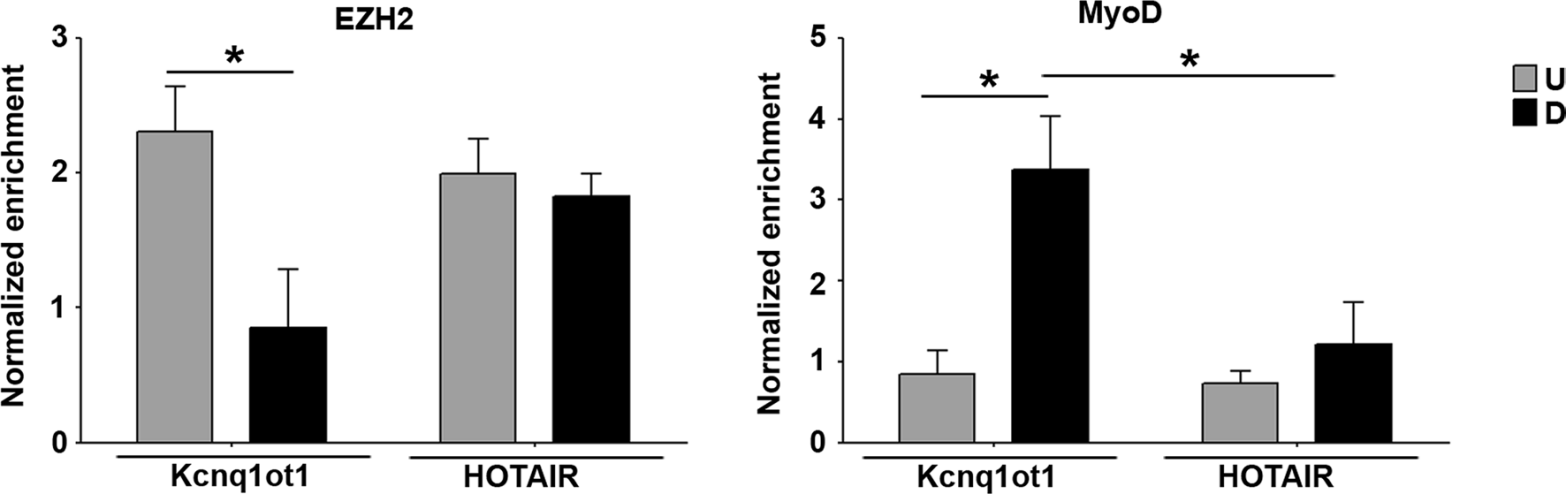
Kcnq1ot1 interacts with EZH2 in undifferentiated and with MyoD in differentiated cells. Cell extracts of undifferentiated (*U*) and differentiated (*D*) C2.7 muscle cells were immunoprecipitated using anti-EZH2 or anti-MyoD antibodies. The immunopurified materials were subjected to RT-qPCR with specific primers for Kcnq1ot1 and HOTAIR transcripts. Values, relative to the mean of three and four independent experiments performed for EZH2 and MyoD, respectively, were expressed as fold enrichment respect to IgG. Statistical significance: *p* < 0.05 (*)

Although the decrease in EZH2 interaction with *p57i* could be explained by the reduction of the enzyme levels known to occur during muscle differentiation [49, 54], we noticed that EZH2 binding is maintained at *p57* promoter, where MyoD does not bind. The observation that the binding of MyoD and EZH2 to *p57i* are mutually exclusive raises the suggestion that MyoD itself may play a direct role in EZH2 dynamics at this region. It has been previously shown that MyoD is capable to functionally interact with ribonucleoprotein complexes, as demonstrated for the non-coding RNA steroid receptor activator SRA [55] and for the muscle-specific LncRNA Linc-RAM [56]. To verify whether MyoD physically interacts with Kcnq1ot1 during differentiation, we performed RNA immunoprecipitation (RIP) assays for MyoD as well as for EZH2. The amount of Kcnq1ot1 transcript associated with either protein was analyzed by RT-qPCR. As shown in Fig. 8, and as already observed in placenta, EZH2 interacts with Kcnq1ot1. However, this interaction, unlike we observed for HOTAIR, another LncRNA known to associate with EZH2 [57], decreases upon differentiation. In contrast, MyoD specifically interacts with Kcnq1ot1, but not with HOTAIR, and only in differentiated cells. The specificity of the interaction between Kcnq1ot1 and MyoD was further verified by an additional RIP assay for the histone demethylase LSD1/KDM1A, known to interact with HOTAIR but not with Kcnq1ot1 [31, 58]. As shown in Additional file 8, the result confirmed that even in differentiated C2 cells, HOTAIR, but not Kcnq1ot1, co-immunoprecipitates with LSD1. These findings insinuate that MyoD, by interacting with Kcnq1ot1 at the maternal intragenic region, could interfere with EZH2 binding, therefore contributing to the chromatin changes that promote *p57* upregulation.

## Discussion

Kcnq1ot1 is a well-recognized player of the machinery that participates in the silencing of paternal *p57* during imprinting. The present work suggests that Kcnq1ot1, similarly to CTCF and KvDMR1, exerts a repressive function also on maternal *p57*, highlighting an additional and unexpected level of *p57* regulation in muscle cells. It is important to notice that in this experimental setting Kcnq1ot1 depletion leads to the upregulation of the maternal active allele but not to the re-expression of the paternal imprinted allele. A possible explanation for the maintenance of imprinting after siRNA treatment could be that the transcriptional silencing and the stable repression of the paternal *Cdkn1c/Kcnq1* locus is reinforced by the cooperation of more than one silencing mechanism. In fact, the establishment and maintenance of the imprinting involve not only the Kcnq1ot1-mediated recruitment of repressive chromatin modifiers, but also the CTCF-mediated enhancer-blocking activity and the chromatin looping. Moreover, despite most evidence suggests that Kcnq1ot1 exerts the silencing activity through its RNA product [23, 36, 59], some results support a model in which it is the act of *Kcnq1ot1* transcription, preserved in knockdown assays, and not the RNA product per se, that plays a role in the imprinting process [60]. Consistent with our finding, it was reported that the depletion of Kcnq1ot1, through short hairpin RNAs, did not cause loss of imprinting in mouse stem cells [36].

Another interesting question raised by our results is how Kcnq1ot1, which is assumed to be paternally expressed in every tissue and to act *in cis*, can regulate maternal *p57*. A maternal Kcnq1ot1 isoform, initiated from an alternative promoter located downstream of the hypermethylated CpG island, and thus escaping silencing, was described in heart tissue [61]. However, this isoform did not affect maternal *p57* expression, probably because it is non-functional due to the lack of the silencing domain, located at the 5′ of the transcript. Although it is possible that some maternally expressed isoform exists also in muscle cells, the location of the primers we used for allele-specific analysis, allows us to exclude the presence of maternal Kcnq1ot1 transcripts including the silencing domain, both in undifferentiated and differentiated cells. An additional and attractive hypothesis is that paternal Kcnq1ot1 could interact with and regulate maternal *p57* through a mechanism involving allele proximity. In this regard, homologous pairing in somatic cells has emerged as a phenomenon more widespread than previously believed [62, 63]. Remarkably, one of the chromosomal regions that were reported to undergo frequent pairing in embryonic stem cells is just the *Cdkn1c/Kcnq1* domain [63]. The significance of allelic interactions in gene regulation, as well as how the homologous regions come together, has not been established yet. However, it is reasonable to expect that the allele proximity creates the opportunity for several types of trans-regulatory interactions, including those involving cis-acting LncRNAs.

Regardless of the *in cis* or *in trans* activity, the results obtained by ChOP assays prove that Kcnq1ot1 interacts with chromatin at an intragenic region of *p57*, not only on the paternal but also on the maternal allele. Importantly, an evident accumulation of EZH2 and H3K27me3 is present at the same region, in particular on the maternal allele. The observation that the levels of EZH2 and H3K27me3 at this region decrease after Kcnq1ot1 depletion supports the conclusion that the LncRNA plays an important role in directing the chromatin modification. These results suggest that a functional interaction between Kcnq1ot1 and PRC2 at the maternal *p57* gene, and in particular at the intragenic region, participates in the repression of *p57* expression in muscle cells. Histone and DNA modifications are well characterized for their regulatory effects on promoter and enhancer regions. However, also gene bodies seem to be the target of epigenetic modifications, even though their function remains unclear. It is commonly believed that repressive modifications, in particular DNA methylation, at intragenic regulatory elements may serve to prevent the occurrence of transcription initiation from non-canonical promoters [64]. More recently, some evidence is emerging that epigenetic modifications at intragenic elements can affect the arrest or release of transcriptional elongation, in this way regulating total mRNA levels and/or alternative splicing through a co-transcriptional mechanism [65, 66]. Interestingly, it has been reported that the decrease in H3K27me3 at some intragenic regions of the muscle-specific genes *Myogenin* and *Muscle creatine kinase* [67] and of a number transforming growth factor β-responsive genes [68] are functionally correlated with the progression of RNA polymerase II through their gene bodies and with their increased expression. Previous results from our laboratory, based on Norther blot, RT-PCR and Western blot analysis, would suggest that the upregulation and downregulation of *p57* in muscle cells concern a single and canonical isoform of p57 mRNA, thus rendering unlikely that the accumulation of H3K27me3 at the newly identified regulatory region may act by causing the arrest of transcriptional elongation or by promoting an alternative splicing event. More work is required not only to definitively clarify this point but also to determine whether the *p57i* may nucleate additional epigenetic modifications and/or promote changes of the three-dimensional architecture of the locus, resulting in transcriptional repression.

**Fig. 9:**
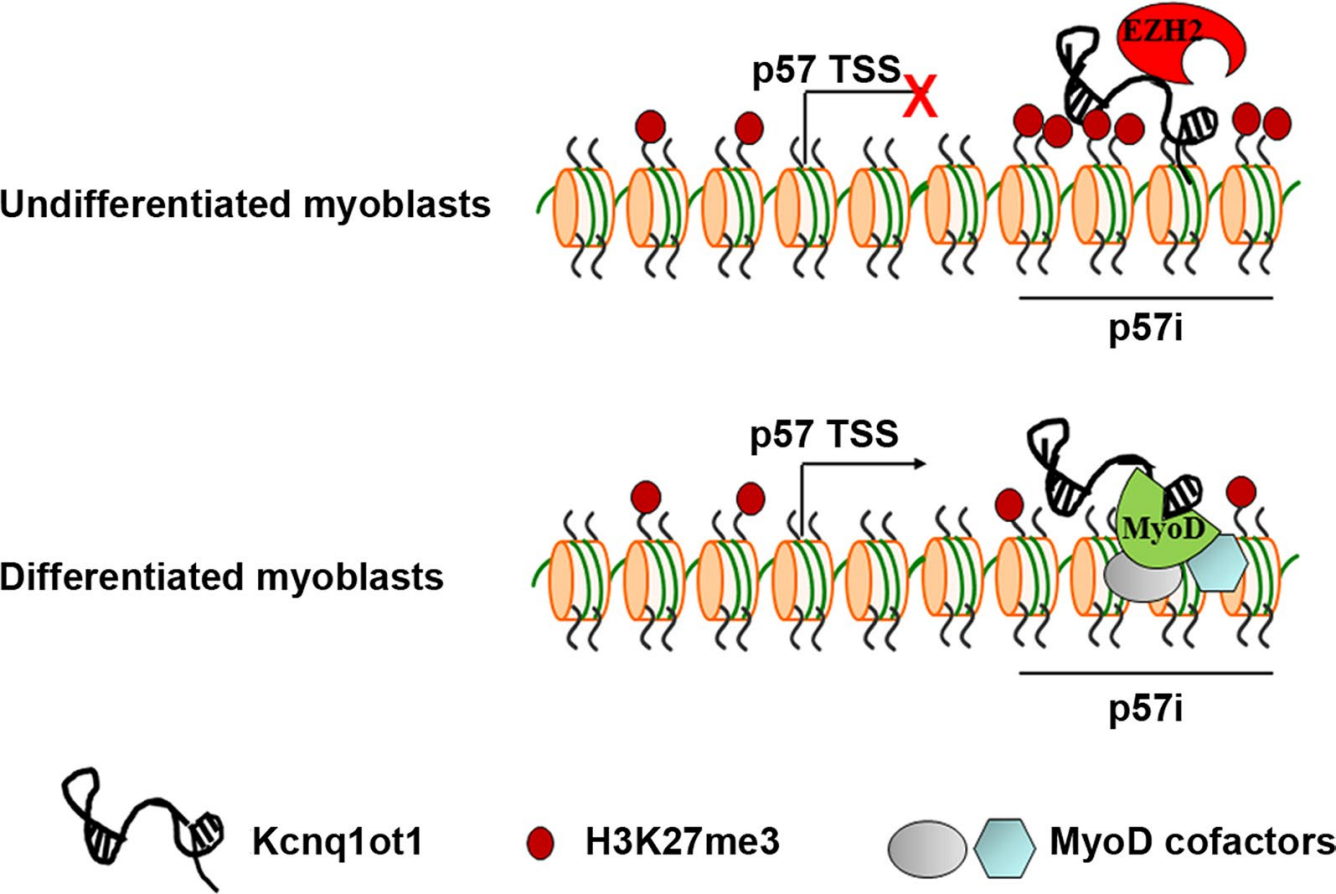
Schematic model of the mechanism by which Kcnq1ot1 regulates *p57* expression during muscle differentiation. In undifferentiated cells Kcnq1ot1 recruits EZH2 and promotes H3K27me3 accumulation on the *p57* intragenic region (*p57i*). Upon differentiation stimuli, the decrease in EZH2 binding and H3K27me3 levels allows the interaction of MyoD and, conceivably, other cofactors with the same region and with Kcnq1ot1, leading to the induction of *p57* expression

Importantly, Kcnq1ot1 remains bound to p57i, while EZH2 and H3K27 levels decrease upon differentiation stimuli, suggesting that the scaffold function of the LncRNA is modulated during differentiation. A dynamic association of Kcnq1ot1 with its interacting partners has been previously proposed to explain the lineage-specific silencing of some imprinted genes [23]. Remarkably, MyoD binding occurs to the same *p57i* bound by Kcnq1ot1, but only after differentiation. The simplest interpretation of these results is that the decrease in EZH2 levels in differentiated cells accounts for the decreased H3K27me3 accumulation, the increased MyoD binding to the E-boxes, and the consequent recruitment of the multiple chromatin modifiers and remodelers engaged by MyoD [69] (see the model depicted in Fig. 9). However, our observation that during differentiation EZH2 levels do not decrease at *p57* promoter, where MyoD does not bind, could indicate that MyoD and EZH2 mutually interfere with each other at *p57i*. A suggestive hypothesis is that MyoD, by physically interacting with Kcnq1ot1, may induce a conformational change of the LncRNA, which could reduce its affinity for EZH2, resulting in the release of repression. The involvement of both ubiquitous and muscle-specific LncRNAs, in the processes of skeletal muscle differentiation and regeneration, is becoming increasingly evident [70–72]. However, the precise function of these LncRNAs in gene regulation is not yet completely clear. The ability of MyoD to interact with regulatory non-coding RNAs, as reported for SRA and Linc-RAM in previous works [55, 56] and as shown for Kcnq1ot1 in the present one, could represent a more widespread phenomenon in the strategy by which the myogenic factor controls muscle differentiation, expanding our understanding of the biology of LncRNAs in the muscle system.

It is worth mentioning that *Kcnq1ot1* is over-expressed in different cancer types, such as colorectal carcinoma [73], glioma [37], lung adenocarcinoma [38] and hepatoma [74]. Moreover, the knockdown of Kcnq1ot1 exerts a tumor-suppressive effect in some of these cancers [37, 38]. How increased *Kcnq1ot1* levels contribute to carcinogenesis is still unknown but it is plausible that the oncogenic function of the LncRNA is mediated, at least in part, by the repression of the maternal *p57* allele, frequently silenced in cancer cells. It is important to point up that, in colorectal cancer cell lines, *Kcnq1ot1* is monoallelically over-expressed [73], thus indicating that the observed effects do not result from loss of imprinting, but instead, may involve a functional interaction between Kcnq1ot1 and *p57* similar to the one that we have highlighted in muscle cells.

## Conclusions

In summary, this work not only highlights an additional mechanism involved in fine tuning the expression of *p57* during muscle differentiation and, possibly in other physiological and pathological processes, but also discloses an imprinting-independent function of Kcnq1ot1, aimed at regulating the maternal, non-imprinted *p57* allele. Moreover, it reveals that Kcnq1ot1 functionally interacts with a tissue-specific member of the bHLH family of transcription factors, adding new insight into the regulatory potential of such a mysterious LncRNA.

A more detailed knowledge of the complex roles of Kcnq1ot1 and of chromatin modifications at the multiple *p57* regulatory regions not only will further clarify the molecular mechanisms underlying the tight and fine regulation of *p57* expression in developing tissues and its silencing in over-growth diseases and cancer, but also will provide a tool for devising strategies aimed at restoring the expression of the CDK inhibitor.

## Methods

### Cell cultures

Mouse polymorphic fibroblasts (C57BL/6 × SD7) and C2.7 muscle cells were grown in Dulbecco’s modified Eagle’s medium (DMEM) (Gibco) supplemented with 10% fetal bovine serum (FBS) (Gibco). To induce MyoD activity and differentiation, cells were shifted to differentiation medium (DMEM supplemented with 0.5% FBS) and collected 24 or 48 h later, as indicated. Production of MyoD-expressing retrovirus and retroviral infections were performed as previously described.

### ChIP assays

ChIP assays were carried out as previously described using 150 µg of chromatin for EZH2 and MyoD, and 50 µg of chromatin for modified histones. In brief, chromatins were immunoprecipitated with anti-MyoD antibody ([sc-760] Santa Cruz Biotechnology), anti-EZH2 antibody (39901, Active Motif), anti trimethyl-histone H3K27 (07-449, Millipore), anti trimethyl-histone H3K4 (07-473, Millipore), normal rabbit (Millipore, 12-370) or normal mouse IgG (Millipore, 12-371). qPCR analysis was performed in triplicate for each of three independent experiments, using 5 ng of DNA, Go Taq qPCR Master Mix (Promega) and the following primer pairs:

*p57* prom: F: 5′5′ACTGAGAGC-AAGCGAACAGG-3′
  R: 5′ACCTGGCTGATTGGTGATGG-3′;
Maternal *p57*i: F: 5′-CAGATCTGACCTCAGACCCA-3′
  R: 5′-CCTGTTCCTCGCCGTCCC-3′;
Paternal *p57*i: F: 5′CAGATCTGACCTCAGACCCG-3′
  R: 5′-GACCTGTTCCTCGCCATCCT-3′;
*β*-*globin* prom: F: 5′-GAAGCCTGATTCCGTAGAGC-3′
  R: 5′-CAACTGATCCTACCTCACCTTATATGC-3′;
*β*-*Actin* prom: F: 5′-GTGACATCCACACCCAGAGG-3′
  R: 5′-GAATAGCCTCCGCCCTTG-3′;
*Amylase* prom: F: 5′-TCAGATGGGAGGACTGCTATTGT-3′
  R: 5′-GCTCACATTCCTTGGCAATATCA-3′;
*Albumin* prom: F: 5′-AATCGTCTTTGAGGCACCAG-3′
  R: 5′-GCTCAATCTTCCCAAACAGG-3′;
*Timm* prom: F: 5′-ACGGATGTGGCCCTTCTGGCT-3′
  R: 5′-CCGCTGCGAAACGCCCACAA-3′;
*p57i*: F: 5′-AACTTCCAGCAGGATGTGCC-3′
  R: 5′-CATCCACTGCAGACGACCAG-3′

### Gene expression analysis

Total RNA was extracted using the High Pure RNA Isolation Kit (Roche) and 1 µg of total RNA was reverse-transcribed using the iScript cDNA Synthesis Kit (Bio-Rad). cDNA obtained was diluted and analyzed through qPCR using the following sets of primers:

p57: F: 5′-AACTTCCAGCAGGATGTGCC-3′
  R: 5′-CATCCACTGCAGACGACCAG-3′
Myogenin: F: 5′-GTCTCTTCCTGAAGCCAGTTGCG-3′
  R: 5′-TGCAAATGCTTGGCCCCCAGAG-3′
Kcnq1ot1: F: 5′-TTCTGGAGGCGATTGAGGC-3′
  R: 5′-AGCAACCAGAACCAGGTGAGAG-3′
Maternal p57: F: 5′-CAGATCTGACCTCAGACCCA-3′
  R: 5′-CCTGTTCCTCGCCGTCCC-3′
Paternal p57: F: 5′CAGATCTGACCTCAGACCCG-3′
  R: 5′-GACCTGTTCCTCGCCATCCT-3′
Maternal Kcnq1ot1: F: 5′-ACTCGGAATTCAGGTGTGGG-3′
  R: 5′-GGTTGGAGGTCACCACAACAT-3′
Paternal Kcnq1ot1: F: 5′-ACTCGGAATTCAGGTGTGGA-3′
  R: 5′-GGTTGGAGGTCACTACAACAT-3′
p21: F: 5′-TGGACATGGTGCCTGTGGCTCT-3′
  R: 5′-AGCAGCCGAGAGGTGTGAGC-3′
Kcnq1: F: 5′-TGAGAAAGATGCGGTGAACG-3′
  R: 5′-GCGTAGCTGCCAAACTCGAT-3′
Tbp: F: 5′-GGCGGTTTGGCTAGGTTT-3′
  R: 5′-GGGTTATCTTCACACACCATG-3

### RNA interference

8 × 10^5^ C2.7 muscle cells were transfected using a mixture of four chemically synthesized siRNAs duplexes (Kcnq1ot1 MOUSE SMARTpool Dharmacon) and with a non-targeting pool (Lincode Non-targeting Control siRNAs Dharmacon) as negative control, at a final concentration of 100 nM. *Kcnq1ot1* expression levels were analyzed 24 h after transfection in proliferation conditions, while 24 h after the shift to differentiation conditions (DMEM containing 0.5% FCS) samples were collected to analyze *p57* and *p21* expression levels.

Polymorphic fibroblasts (C57BL/6 × SD7) expressing MyoD were transfected 48 h after infection, with 200 nM siRNAs. Samples were collected 24 h after transfection to check the reduction of *Kcnq1ot1* expression and 24 h after the shift to differentiation medium to determine *p57* and *p21* expression levels.

### ChOP assays

ChOP assays were carried out according to the published protocol [23, 75] with some modifications. In brief, approximately 10 × 10^6^ cells were fixed with 1% formaldehyde for 10 min at 37 °C. Glycine was added to a final concentration of 0,125 M in phosphate-buffered saline (PBS) for 5 min at 4 °C and 5 min at room temperature with gentle shaking. Cells were washed with cold PBS, scraped and nuclei were isolated in buffer A (3 mM MgCl2, 10 mM Tris–Hcl (pH 7.4), 10 mM NaCl, and 0.5% NP-40). After centrifugation and washing once in an equal volume of buffer A, nuclei were lysed in buffer B (50 mM Tris–HCl [pH 7.9], 10 mM EDTA, 0,2 mM PMSF, 1%SDS, supplemented with protease inhibitors and 100 U/ml RNase inhibitor), and incubated on ice for 10 min. An equal volume of buffer C (15 mM Tris–HCl [pH 7.9], 150 mM NaCl, 1 mM EDTA, 1% Triton-X-100, 0,2 mM PMSF, protease and RNase inhibitors) was added, and nuclear chromatin was sonicated to obtain fragment sizes ranging from 300 to 700 bp in length. Chromatin was quantified for DNA, and 150 μg aliquots were prepared for each sample. Protein A Sepharose was blocked in an equal volume of buffer D (15 mM Tris–HCl [pH 7.9], 150 mM NaCl, 1 mM EDTA, 0,5% NP-40, 0,2 mM PMSF and proteinase inhibitors) containing 400μg/ml yeast RNA and 800 μg/ml of bovine serum albumin (BSA) for 4 h. Beads were washed three times with buffer D and incubated overnight at 4 °C with 5 μg of anti-biotin antibody (ab66-43-100, Abcam). The sonicated chromatin was incubated overnight at 4 °C with 25 pmol of biotin-labeled oligo antisense to Kcnq1ot1 (biotin 5′-CCAAAAGAACTGTGGACAAATATGCTGAGGCTG-3′), or biotin-labeled Scrambled oligo (biotin 5′-GCCAAGTGTTAAAGGCCAAACTACGTTGAGAGA-3′), and incubated overnight at 4 °C. 50 μl of beads with bound antibody was added to samples containing the oligonucleotides and incubated for 4 h at 4 °C. Before washing, the supernatant of the scrambled samples was taken as Input. Each sample was washed twice with Low Salt buffer (SDS 0.1%, Triton-X-100, EDTA 2 mM, Tris–HCl 20 mM pH = 8.1, NaCl 150 mM) and with High Salt buffer (SDS 0.1%, Triton-X-100, EDTA 2 mM, Tris–HCl 20 mM pH = 8.1, NaCl 500 mM); once time with LiC Buffer (LiCl 0,25 M, NP40 1%, deoxycholate 1%, EDTA 1 mM, Tris–HCl10 mM pH = 8,1) and TE Buffer (Tris-HCl 10 mM, EDTA 1 mM pH = 8). Samples were eluted with 300μl of Elution Buffer (TE 1x, SDS 0.5%), treated with 1 μg of RNAse A (Sigma-Aldrich) for 10 min at room temperature and with and 240 μg of Proteinase K (Sigma-Aldrich) for 5 h at 62 °C. The DNA was extracted with phenol–chloroform, precipitated with isopropanol and re-suspended in 50 μl of nuclease-free water (Promega). After the immunoprecipitation, the DNA concentration of each sample was measured. The enrichment of Kcnq1ot1-interacting genomic DNA regions was analyzed by qPCR using the following primers:

Maternal KvDMR1: F: 5′-ACTCGGAATTCAGGTGTGGG-3′
  R: 5′-GGTTGGAGGTCACCACAACAT-3′
Paternal KvDMR1: F: 5′-ACTCGGAATTCAGGTGTGGA-3′
  R: 5′-GGTTGGAGGTCACTACAACAT-3′
*p57* promoter: F: 5′-ACTGAGAGC-AAGCGAACAGG-3′
  R: 5′-ACCTGGCTGATTGGTGATGG-3′
KvDMR1: F: 5′-GCACAAGTCGCAAGTCCGCG-3′
  R: 5′-ATGGAGCCCAGCCGCGAAAG-3′
Maternal *p57*i: F: 5′-CAGATCTGACCTCAGACCCA-3′
  R: 5′-GACCTGTTCCTCGCCATCCT-3′
Paternal *p57*i: F: 5′AACTTCCAGCAGGATGTGCC-3′
  R: 5′-GACCTGTTCCTCGCCATCCT-3′
*p57i*: F: 5′-AACTTCCAGCAGGATGTGCC-3′
  R: 5′-CATCCACTGCAGACGACCAG-3′
*Dppa2* prom: F: 5′-TGCCCTGGATTTAAAACGTC-3′
  R: 5′-CGAGCTTTGTCCTCCTGGTA-3′
*Nap1l4* prom: F: 5′-AGGTGTTGGGATTGAAGGTG-3′
  R: 5′-CACCCAATACAAAGGCTGCT-3′

### RIP assays

Undifferentiated and differentiated C2.7 muscle cells were harvested in PBS supplemented with 0,2 mM PMSF. Nuclei were isolated with buffer A (20 mM Tris–HCl [pH 8.0], 10 mM NaCl, 3 mM MgCl2, 0,1% NP40, 10% glycerol, 0,2 mM EDTA, with 0,4 mM PMSH, proteinase inhibitors and RNAse Inhibitors [100U/μl]), incubated at 4 °C for 30 min. Nuclei were extracted with NT2 buffer (50 mM Tris–HCl [pH 7.4], 150 mM NaCl, 1 mM MgCl2, 0,5% NP-40, 20 mM EDTA, 0,4 mM PMSF, 1 mM DTT, and proteinase and RNase Inhibitors). Nuclear protein extracts were quantified and 1,5 mg of protein were used for each sample. 100μl of protein A Sepharose were incubated with 5 μg of anti-MyoD antibody (sc-760, Santa Cruz), anti-EZH2 antibody(39901, Active Motif), anti-LSD1 antibody (ab 17721 Abcam), normal rabbit IgG ([12-370] Merk Millipore) or normal mouse IgG ([12-371] Merk Millipore) for 6 h at 4 °C. Lysate pre-clearing was performed using 150μl of protein A Sepharose and keeping samples for 6 h at 4 °C. 100 μl of beads with bound antibody were added to pre-cleared samples and incubated overnight at 4 °C. Before washing, the supernatant of the IgG sample was taken as Input. Each sample was washed four times with 500 μl of NT2 buffer, treated with 10U of DNase (Roche), RNA was extracted with Trizol Reagent (Invitrogen) and precipitated overnight with isopropanol. RNA was re-suspended in 25 μl of nuclease-free water (Promega), and Kcnq1ot1 enrichment was analyzed by RT-qPCR using the following primers:

Kcnq1ot1: F: 5′-TTCTGGAGGCGATTGAGGC-3′
  R: 5′-AGCAACCAGAACCAGGTGAGAG-3′
HOTAIR: F: 5′-GCGCCAACGTAGACCAAAAG-3′
  R: 5′-TACCGATGTTGGGGACCTCT-3′

### Statistical analysis

For statistical analysis, comparisons were performed using parametric paired Student’s *t* test. Statistical significance is shown as *p* < 0.05 (*) or *p* < 0.01 (**) or *p* < 0.001 (***).

## Additional files

**Additional file 1.** *Kcnq1ot1 knockdown boosts p57 induction in MyoD*-*converted fibroblasts.* Polymorphic fibroblasts (C57B/6 female × SD7 male) infected with the MyoD retroviral vector were transfected with Kcnq1ot1 or control siRNAs and analyzed by RT-qPCR for *p57* and *p21* expression 24 h after the shift to differentiation medium. Values, relative to those of Tbp RNA, are the mean ± SEM of three independent experiments. Statistical significance: *p* value < 0.05 (*).

**Additional file 2.** Kcnq1ot1 knockdown affects maternal but not paternal p57 expression. RNAs from siCTR and siKcnq1ot1 samples (prepared as described in Additional file 1) were amplified by RT-PCR with primers surrounding the single nucleotide polymorphism. Maternal and paternal cDNAs were distinguished by RFLP analysis of previously described polymorphic restriction sites; ND (Non-digested samples) indicates the electrophoretic mobility of the undigested p57 paternal-specific amplicon, while AVA I indicates the electrophoretic mobilities of the AVA I-digested maternal-specific fragments; the RFLP analysis shown represents one of three independent experiments.

**Additional file 3.** Kcnq1ot1 expression does not decrease during differentiation. RT-qPCR analysis of Kcnq1ot1, expression in undifferentiated (*U*) and differentiated (*D*) C2.7 muscle cells; *p57* and the muscle-specific gene *Myogenin* (*MyoG*) were used to follow the differentiation process. Values, relative to those of Tbp RNA, are the mean ± SEM of four independent experiments. Statistical significance: *p* value < 0.05 (*); *p* value < 0.01 (**).

**Additional file 4.** Differential epigenetic status of the maternal and paternal p57 intragenic regions. Left: Allele-specific ChIP-qPCR analysis of H3K4me3 accumulation at Maternal and Paternal *p57* intragenic regions (M-*p57i* and P-*p57i*, respectively) in polymorphic fibroblasts; *Amylase* promoter (*Amy* p) was used as negative control. Values are the mean ± SEM of three independent experiments performed and were expressed as percentages of Input. Statistical significance: *p* value < 0.05 (*). Right: qPCR analysis of the MeDIP assays performed in polymorphic fibroblasts (C57B/6 female × SD7 male) using allele-specific primers for the *p57* intragenic region (M-*p57i* and P-*p57i*, respectively). *Translocase of inner mitochondrial membrane 17* promoter (*Timm* p) was used as a negative control. The results shown represent one of two independent experiments performed. Values were expressed as percentages ± SEM of Input DNA for each sample analyzed in triplicate.

**Additional file 5.** Verification of Kcnq1ot1 depletion in cells used for the ChIP assays reported in Fig. 6. C2.7 myoblasts were transfected with Kcnq1ot1 siRNAs as in Fig. 1a and analyzed by RT-qPCR for Kcnq1ot1 RNA levels in siCTR and siKcnq1ot1 samples. Values were normalized to Tbp RNA levels and expressed as percentages of the control. Results are the mean ± SEM of three independent experiments. Statistical significance: *p* value < 0.001 (***)

**Additional file 6.** *H3K27me3 association to the p57 intragenic region decreases after Kcnq1ot1 depletion*. Polymorphic fibroblasts (C57B/6 female × SD7 male) infected with the MyoD retroviral vector were transfected with Kcnq1ot1 or control siRNAs as in Fig. 2a and analyzed by ChIP-qPCR for H3K27 association to the maternal *p57* intragenic region (M-*p57i*), *β*-*Globin* promoter (*β*-*Glob* p) used as an invariant control and β-Actin promoter (β-Act p) as a negative control. Values obtained are expressed as percentages of Input chromatin and normalized to those of *Albumin* promoter, used as an additional invariant control. The results shown represent one of two independent experiments and error bars represent the mean ± SEM of each sample analyzed in triplicate.

**Additional file 7.** H3K27me3 association to the p57 intragenic region decreases during differentiation. ChIP-qPCR analysis of H3K27me3 association to the *p57* intragenic region (*p57i*) and *p57* promoter *(p57* p) in undifferentiated (U) and differentiated (D) C2.7 muscle cells. *β*-*Actin* promoter (*β*-*Act* p) was used as a negative control. Values obtained were expressed as percentages of Input chromatin and normalized to those of *Albumin* promoter, used as an invariant control. The results are the mean ± SEM of three independent experiments. Statistical significance: *p* value < 0.05 (*).

**Additional file 8.** Kcnq1ot1 and HOTAIR are differentially associated with LSD1. Cell extracts of differentiated C2.7 muscle cells were immunoprecipitated using anti-LSD1 antibody or control IgG. Immunopurified materials were subjected to RT-qPCR with specific primers for Kcnq1ot1 and HOTAIR transcripts. Values, relative to a representative experiment, were expressed as fold enrichment respect to IgG.

**Additional file 9.** Additional methods.
