# Supplementary figures and images for: The long non-coding RNA Kcnq1ot1 controls maternal p57 expression in muscle cells by promoting H3K27me3 accumulation to an intragenic MyoD-binding region
Source: Epigenetics Chromatin. 2019 Jan 16;12:8. doi: 10.1186/s13072-019-0253-1 (PMC6334472; doi:10.1186/s13072-019-0253-1)

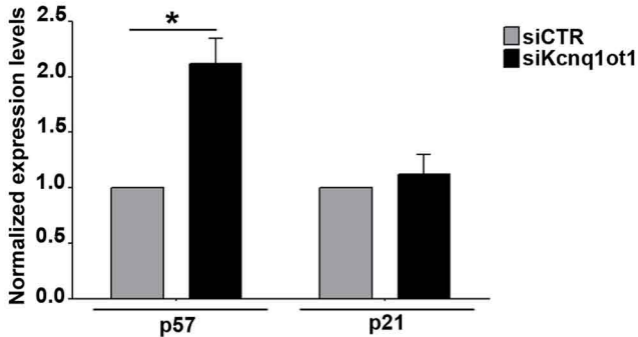

Supplement: Supplementary file 1 — Additional file 1. Kcnq1ot1 knockdown boosts p57 induction in MyoD-converted fibroblasts. Polymorphic fibroblasts (C57B/6 female × SD7 male) infected with the MyoD retroviral vector were transfected with Kcnq1ot1 or control siRNAs and analyzed by RT-qPCR for p57 and p21 expression 24 h after the shift to differentiation medium. Values, relative to those of Tbp RNA, are the mean ± SEM of three independent experiments. Statistical significance: p value < 0.05 (*). [file 13072_2019_253_MOESM1_ESM.pdf]

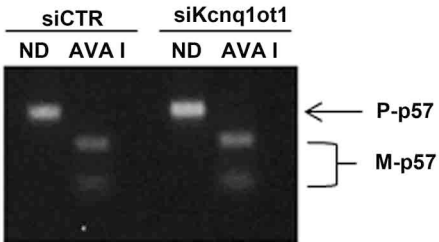

Supplement: Supplementary file 2 — Additional file 2. Kcnq1ot1 knockdown affects maternal but not paternal p57 expression. RNAs from siCTR and siKcnq1ot1 samples (prepared as described in Additional file 1) were amplified by RT-PCR with primers surrounding the single nucleotide polymorphism. Maternal and paternal cDNAs were distinguished by RFLP analysis of previously described polymorphic restriction sites; ND (Non-digested samples) indicates the electrophoretic mobility of the undigested p57 paternal-specific amplicon, while AVA I indicates the electrophoretic mobilities of the AVA I-digested maternal-specific fragments; the RFLP analysis shown represents one of three independent experiments. [file 13072_2019_253_MOESM2_ESM.pdf]

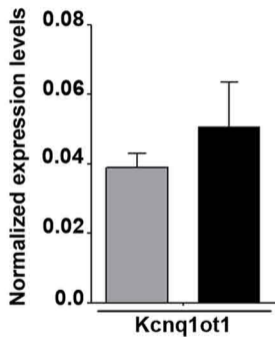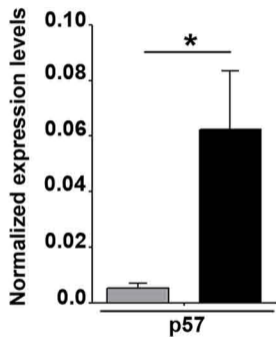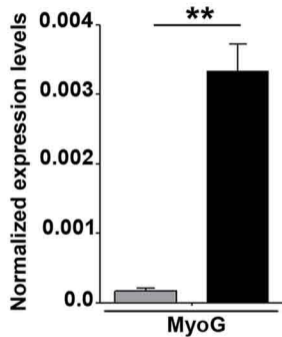

■ U ■ D

Supplement: Supplementary file 3 — Additional file 3. Kcnq1ot1 expression does not decrease during differentiation. RT-qPCR analysis of Kcnq1ot1, expression in undifferentiated (U) and differentiated (D) C2.7 muscle cells; p57 and the muscle-specific gene Myogenin (MyoG) were used to follow the differentiation process. Values, relative to those of Tbp RNA, are the mean ± SEM of four independent experiments. Statistical significance: p value < 0.05 (*); p value < 0.01 (**). [file 13072_2019_253_MOESM3_ESM.pdf]

**a**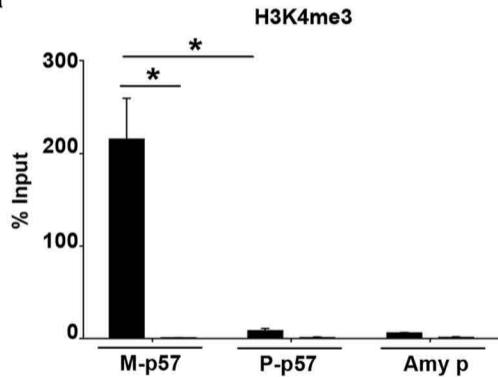

■ IP    □ IgG

**b**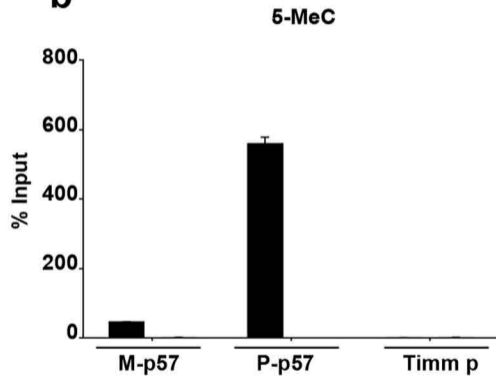

Supplement: Supplementary file 4 — Additional file 4. Differential epigenetic status of the maternal and paternal p57 intragenic regions. Left: Allele-specific ChIP-qPCR analysis of H3K4me3 accumulation at Maternal and Paternal p57 intragenic regions (M-p57i and P-p57i, respectively) in polymorphic fibroblasts; Amylase promoter (Amy p) was used as negative control. Values are the mean ± SEM of three independent experiments performed and were expressed as percentages of Input. Statistical significance: p value < 0.05 (*). Right: qPCR analysis of the MeDIP assays performed in polymorphic fibroblasts (C57B/6 female × SD7 male) using allele-specific primers for the p57 intragenic region (M-p57i and P-p57i, respectively). Translocase of inner mitochondrial membrane 17 promoter (Timm p) was used as a negative control. The results shown represent one of two independent experiments performed. Values were expressed as percentages ± SEM of Input DNA for each sample analyzed in triplicate. [file 13072_2019_253_MOESM4_ESM.pdf]

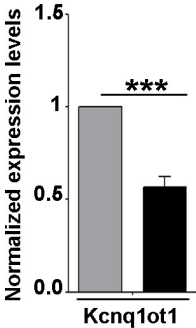

Supplement: Supplementary file 5 — Additional file 5. Verification of Kcnq1ot1 depletion in cells used for the ChIP assays reported in Fig. 6. C2.7 myoblasts were transfected with Kcnq1ot1 siRNAs as in Fig. 1a and analyzed by RT-qPCR for Kcnq1ot1 RNA levels in siCTR and siKcnq1ot1 samples. Values were normalized to Tbp RNA levels and expressed as percentages of the control. Results are the mean ± SEM of three independent experiments. Statistical significance: p value < 0.001 (***) [file 13072_2019_253_MOESM5_ESM.pdf]

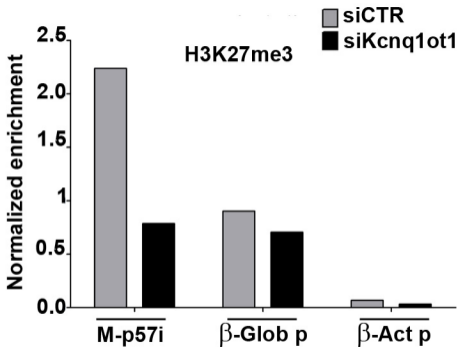

Supplement: Supplementary file 6 — Additional file 6. H3K27me3 association to the p57 intragenic region decreases after Kcnq1ot1 depletion. Polymorphic fibroblasts (C57B/6 female × SD7 male) infected with the MyoD retroviral vector were transfected with Kcnq1ot1 or control siRNAs as in Fig. 2a and analyzed by ChIP-qPCR for H3K27 association to the maternal p57 intragenic region (M-p57i), β-Globin promoter (β-Glob p) used as an invariant control and β-Actin promoter (β-Act p) as a negative control. Values obtained are expressed as percentages of Input chromatin and normalized to those of Albumin promoter, used as an additional invariant control. The results shown represent one of two independent experiments and error bars represent the mean ± SEM of each sample analyzed in triplicate. [file 13072_2019_253_MOESM6_ESM.pdf]

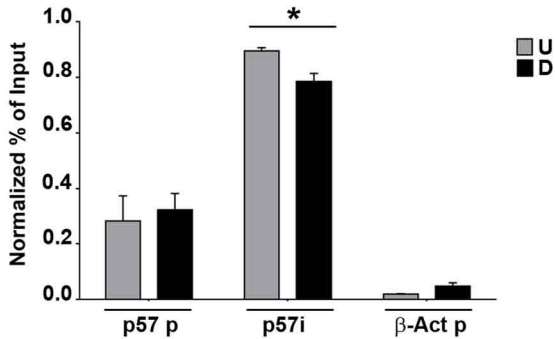

Supplement: Supplementary file 7 — Additional file 7. H3K27me3 association to the p57 intragenic region decreases during differentiation. ChIP-qPCR analysis of H3K27me3 association to the p57 intragenic region (p57i) and p57 promoter (p57 p) in undifferentiated (U) and differentiated (D) C2.7 muscle cells. β-Actin promoter (β-Act p) was used as a negative control. Values obtained were expressed as percentages of Input chromatin and normalized to those of Albumin promoter, used as an invariant control. The results are the mean ± SEM of three independent experiments. Statistical significance: p value < 0.05 (*). [file 13072_2019_253_MOESM7_ESM.pdf]

## LSD1

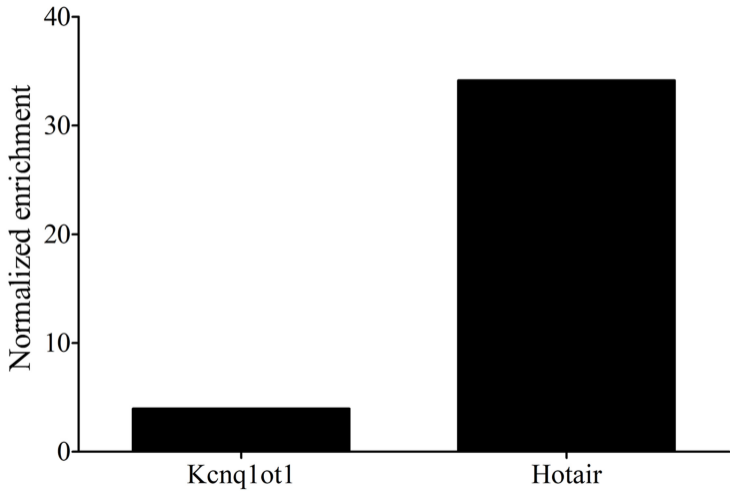

Supplement: Supplementary file 8 — Additional file 8. Kcnq1ot1 and HOTAIR are differentially associated with LSD1. Cell extracts of differentiated C2.7 muscle cells were immunoprecipitated using anti-LSD1 antibody or control IgG. Immunopurified materials were subjected to RT-qPCR with specific primers for Kcnq1ot1 and HOTAIR transcripts. Values, relative to a representative experiment, were expressed as fold enrichment respect to IgG. [file 13072_2019_253_MOESM8_ESM.pdf]
