# Supplementary material for: The long non-coding RNA Kcnq1ot1 controls maternal p57 expression in muscle cells by promoting H3K27me3 accumulation to an intragenic MyoD-binding region
Source: Epigenetics Chromatin. 2019 Jan 16;12:8. doi: 10.1186/s13072-019-0253-1 (PMC6334472; doi:10.1186/s13072-019-0253-1)
Supplement: Supplementary file 9 — Additional file 9. Additional methods. [file 13072_2019_253_MOESM9_ESM.pdf]

ADDITIONAL METHODS

RFLP analysis

To confirm the allele-specificity of *p57* expression, cDNAs from MyoD-infected polymorphic fibroblasts (C57BL/6 x SD7) were amplified by semiquantitave PCR with the following primers:

F: 5’-TTCAGATCTGACCTCAGACCC-3’

R: 5’-AGTTCTCTTGCGCTTGGC-3’.

Maternal and paternal products were distinguished by AVA I (Promega) digestion of a previously described polymorphic restriction site for this enzyme (Battistelli et al., 2014).

Methylated DNA Immunoprecipitation (MeDIP) assays

Genomic DNA was extracted, purified and sonicated in order to obtain fragments of a length in a range of 300-600 bp. 6 µg of sonicated DNA was re-suspended in 500 µL of Ip Buffer [Immunoprecipitation Buffer (NaCl 140mM, Triton X-100 0.05%, sodium phosphate 10mM pH=7)]. The denaturation of DNA was performed 5 minutes at 95 °C and the incubation with 5 µg of mouse anti 5-Methylcytidine antibody (Bi-Mecy 0100, Eurogentec) or with anti normal mouse IgG antibody (12-371, Merk Millipore) over-night at 4°C. 40 µL of protein G magnetic beadswere added to samples and incubated 4 hours at 4 °C. The supernatant of the IgG sample was taken as Input and beads were washed 3 times with Ip Buffer. After washes samples were incubated for 2 hours at 56 °C and overnight at 37°C with proteinase K (Sigma-Aldrich). The extraction of DNA was performed with phenol-chloroform solution, precipitated with ethanol and the pellet of DNA was re-suspended in 50 µl of nuclease-free distilled water**.** qPCR analyses of immunoprecipitated maternal and paternal *p57* intragenic region and, as negative control, for of the *Translocase of inner mitochondrial membrane 17* (*Timm*) were performed each in triplicate using 5 ng of DNA, GoTaq qPCR Master Mix (Promega) using the termocycler “CFX Connect Real Time system” (Bio-Rad) and the following set of primers:

Maternal *p57*i F: 5’- CAGATCTGACCTCAGACCCG-3’
R: 5’- GACCTGTTCCTCGCCATCCT-3’;
Paternal *p57*i F: 5’-AACTTCCAGCAGGATGTGCC-3’
R: 5’-CATCCACTGCAGACGACCAG-3’;
Timm prom: F: 5’-ACGGATGTGGCCCTTCTGGCT-3’
R: 5’-CCGCTGCGAAACGCCCACAA-3’.
